# Associations between characteristics of the home food environment and fruit and vegetable intake in preschool children: A cross-sectional study

**DOI:** 10.1186/1471-2458-11-938

**Published:** 2011-12-16

**Authors:** Rebecca Wyse, Elizabeth Campbell, Nicole Nathan, Luke Wolfenden

**Affiliations:** 1 Faculty of Health, School of Medicine and Public Health, University of Newcastle, c/o Locked Bag 10, Wallsend, NSW 2287, Australia; 2 Hunter Medical Research Institute (HMRI), Newcastle, Australia; 3 Hunter New England Population Health, Hunter New England Area Health Service, Newcastle, Australia

## Abstract

**Background:**

Early childhood is critical to the development of lifelong food habits. Given the high proportion of children with inadequate fruit and vegetable consumption, identification of modifiable factors associated with higher consumption may be useful in developing interventions to address this public health issue. This study aimed to identify the characteristics of the home food environment that are associated with higher fruit and vegetable consumption in a sample of Australian preschool children.

**Methods:**

A cross-sectional telephone survey was conducted with 396 parents of 3 to 5 year-old children attending 30 preschools within the Hunter region, New South Wales, Australia. Children's fruit and vegetable consumption was measured using a valid and reliable subscale from the Children's Dietary Questionnaire. Associations were investigated between children's fruit and vegetable intake and characteristics of the home food environment including parental role-modeling, parental providing behaviour, fruit and vegetable availability, fruit and vegetable accessibility, pressure to eat, family eating policies and family mealtime practices. Characteristics of the home food environment that showed evidence of an association with children's fruit and vegetable consumption in simple regression models were entered into a backwards stepwise multiple regression analysis. The multiple regression analysis used generalised linear mixed models, controlled for parental education, household income and child gender, and was adjusted for the correlation between children's fruit and vegetable consumption within a preschool.

**Results:**

The multiple regression analysis found positive associations between children's fruit and vegetable consumption and parental fruit and vegetable intake (p = 0.005), fruit and vegetable availability (p = 0.006) and accessibility (p = 0.012), the number of occasions each day that parents provided their child with fruit and vegetables (p < 0.001), and allowing children to eat only at set meal times all or most of the time (p = 0.006). Combined, these characteristics of the home food environment accounted for 48% of the variation in the child's fruit and vegetable score.

**Conclusions:**

This study identified a range of modifiable characteristics within the home food environment that are associated with fruit and vegetable consumption among preschool children. Such characteristics could be considered potential targets for interventions to promote intake among children of this age.

## Background

Adequate consumption of fruit and vegetables provides children with essential nutrients for healthy growth and development [1] and may displace the consumption of energy-dense, nutrient-poor foods associated with childhood overweight and obesity [2-6]. Given that childhood diet is a significant determinant of adult diet [7] and higher fruit and vegetable consumption in childhood is associated with decreased risk of adult chronic disease [8,9], the benefits of adequate childhood fruit and vegetable consumption appear to extend throughout the lifespan. Despite this, internationally, a high proportion of children have inadequate fruit and vegetable intake [10,11]. Identifying factors associated with higher childhood fruit and vegetable consumption may assist in the development of interventions to address this public health issue.

Many factors influence the foods that children eat: the demographic and socio-economic characteristics of their families [10,11]; their individual preferences and genetic predispositions [12,13]; psychosocial factors [14]; and characteristics of their environment [15,16]. Given the amount of time children spend in the home, this environment represents a potentially promising setting in which to improve young children's fruit and vegetable consumption. Rosenkranz's ecological model of the home food environment hypothesises that child diet in this setting is influenced by three domains: built and natural environments; political and economic environments; and socio-cultural environments [17]. Of these, those most proximal to a child's life, such as home accessibility and availability of foods (built and natural environments) and parental diet, parenting practices and rules, and family eating patterns (socio-cultural environments) may be most amenable to intervention. As such, research investigating associations between these characteristics of the home environment and children's fruit and vegetable consumption is warranted.

Studies of school-aged children have found parental fruit and vegetable intake and the accessibility and availability of fruit and vegetables in the home [12,15,16] to be consistently associated with children's consumption. However, research involving children of preschool age (children aged 3 to 5 years [18]) is limited. For example, a 2007 systematic review that included environmental correlates of children's fruit and vegetable intake identified just three studies involving children of preschool-age, compared with 30 studies involving children aged 5-18 years [16], while a more recent systematic review only included studies of children aged 6 years and older [15]. The factors influencing dietary habits in early childhood be may distinct from those affecting school-aged children, due to preschoolers' earlier developmental stage and greater dependence on their family [15]. The few studies that have investigated associations between such factors and fruit and vegetable consumption in preschool children found positive associations with parental fruit and vegetable intake [19-21] and parental role-modeling [22]; and negative associations with eating in front of the television [23] and parental pressure to eat [21,23]. However, only a minority of these studies, have used both a comprehensive or validated assessment of child fruit and vegetable consumption and multivariate analyses to isolate the effect of individual variables and control for the influence of socio-demographic characteristics [20,21]. As such, this study sought to address these limitations, and identify characteristics of the home food environment associated with fruit and vegetable consumption in a sample of Australian preschool children.

## Methods

### Design

A cross-sectional survey of parents of preschool-aged children was conducted via Computer Assisted Telephone Interview (CATI).

### Ethical approval

The data for the present study forms the baseline dataset for a cluster randomised controlled trial of a telephone-based intervention to increase fruit and vegetable consumption in preschool children [24]. Ethical approval for the broader trial was obtained from the Human Research Ethics Committees of the University of Newcastle (Ref No. H-2008-0410) and the Hunter New England Area Health Service (Ref No. 08/10/15/5.09).

### Sample

Study participants were parents of 3 to 5 year-old children attending non-government preschools within the Hunter region of New South Wales, Australia. Almost 90% of preschools within New South Wales are either privately run or run by the community [25]. All participants had previously volunteered to participate in a telephone-based randomised controlled trial of a fruit and vegetable intervention [24]. Preschools were ineligible if they provided children with meals, if they catered exclusively for children with special needs, were government preschools (as the conduct of this research was not approved in these institutions) or if they had participated in child healthy eating research projects within the prior six months. Parents were eligible if they resided with their preschool child for at least 4 days per week and were responsible for their child's meals and snacks at least half of the time. If children had dietary restrictions that were incompatible with the Australian Dietary Guidelines for fruit and vegetable consumption (as determined by an Accrediting Practicing Dietitian), their parents were deemed ineligible.

### Recruitment

Recruitment procedures are described in detail elsewhere [24] and are based on a systematic review identifying effective strategies for recruiting parents for study participation through schools [26]. Briefly, all eligible preschools within the study area were invited to participate. At consenting preschools, a research assistant distributed study information and consent forms to parents as they dropped off or picked up their child. Parents indicated their consent by ticking a box on the consent form and returning it to a drop box at the preschool. Recruitment of preschools began in February 2010 and recruitment of parents began in March 2010 and was conducted over a 6-month period. The consent form contained questions about the parent's residential suburb, the child's age, gender, and usual fruit and vegetable consumption (average number of serves per day). In order to assess bias due to selective non-participation, parents who did not wish to participate in the study were encouraged to also complete a consent form with this information and return it to the preschool.

### Data Collection

Consenting parents were contacted to complete a telephone survey via CATI delivered by telephone interviewers experienced in conducting health-related interviews. The survey was conducted from April to October 2010. Parents were instructed to answer with respect to their preschool-aged child. If they had more than one child aged 3 to 5 years, they were instructed to select the child who would have the next birthday.

### Measures

#### Participant characteristics

The survey included items to assess the socio-demographic characteristics of parents and children. Parents were asked their age, gender, highest level of education and annual household income and whether they identified as Aboriginal and/or Torres Strait Islander, and were asked to report their child's date of birth and gender. Items were sourced from population health surveys [27]. Parents were also asked the number of days per week that they resided with their child, and how often they were responsible for providing their child with meals and snacks (always, most of the time, half of the time, seldom, never).

#### Children's fruit and vegetable intake

Participants also completed the fruit and vegetable subscale of the Children's Dietary Questionnaire (CDQ) [28]. This subscale requires parents to report the variety and frequency of fruit and vegetables consumed by their child over the past 24 hours and past 7 days. This tool includes potatoes and sweet potatoes in the assessment of vegetable consumption, but parents are specifically instructed not to include hot chips. Fruit and vegetable juices are excluded from most (3 out of 4) questions assessing the frequency and variety of fruit intake, with the exception of the number of occasions of fruit or vegetable consumption over the past 24 hours. The subscale score ranges from 0 to 28 and a score of 14 or more suggests that the child has intake patterns consistent with fruit and vegetable dietary guidelines [28]. Changes on this score could arise from a range of possible changes to children's fruit and vegetable consumption patterns, for example, a one-point increase could result from eating an additional type of fruit or vegetable, or eating fruit or vegetable at an additional occasion in the previous 24 hours. This subscale has been established as reliable in comparable samples of preschoolers (intracorrelation coefficient = 0.75), and has been established as valid against a 7-day dietary checklist in a sample of school-aged children (Spearman's correlation coefficient = 0.58) [28].

#### Characteristics of the home food environment

Characteristics of the home food environment were assessed within seven domains: Parental role-modeling of fruit and vegetable consumption, pressure to eat, parental provision of fruit and vegetables, fruit and vegetable availability, fruit and vegetable accessibility, mealtime practices and family eating policies. Where possible, items were taken from existing validated measures of the home food environment including The Healthy Home Survey [29], The Child Feeding Questionnaire [30] and The National Nutrition Survey [31]. Where known, the reliability and validity of items are provided alongside each item. Study items with unknown psychometric properties are also identified.

##### a) Parental role-modeling of fruit and vegetable consumption

Items from the National Nutrition Survey were included to assess the average number of serves of fruit and vegetables consumed each day by parents [31]. Answers to these questions have been positively associated with objective biomarkers of fruit and vegetable intake including α-carotene, β-carotene, β-cryptoxanthin, lutein/zeaxanthin and red-cell folate [32]. A lack of existing role-modeling items that were specific to fruit and vegetable consumption, quantitative, and targeted at parents of preschool children led the study team to develop two items to assess this. Specifically, parents were asked separate questions regarding the number of times they had consumed fruits, and the number of times they had consumed vegetables, in front of their child on the previous day. The validity of these items is unknown.

##### b) Pressure to eat

The 'Pressure to Eat' subscale from Birch's Child Feeding Questionnaire was included to measure the extent to which parents try to control the amount and type of food eaten by their child [30]. Scores range from 1 to 5 and a higher score indicates more pressure. The four-item scale has been shown to be internally consistent (Cronbach's alpha = 0.70) and reliability estimates for the four items of this subscale are 0.19-0.52 [30].

##### c) Parental provision of fruit and vegetables

As no items or scales could be identified that measured the extent to which parents provided their children with fruit and vegetables, two questions were developed specifically for this study, and as such their psychometric properties are unknown. Separate questions were asked regarding the number of occasions on the previous day that the parent provided the child with fruits and with vegetables.

##### d) Availability of fruit and vegetables in the home

As no appropriate measure of home fruit and vegetable availability that was brief, suitable for telephone data collection, and appropriate for use within an Australian sample could be sourced, to assess this, parents were read a list of 19 commonly consumed fruits and 24 commonly consumed vegetables from the Children's Dietary Questionnaire. Fruit and vegetables could be available in any form: fresh, tinned, frozen or dried. Parents were asked to identify those that they had in their home at that time and the number of varieties of fruits and vegetables were then summed. The validity and reliability of this item are unknown.

##### e) Accessibility of fruit and vegetables in the home

Accessibility was assessed by asking whether fruit and vegetables in the home were stored in a form that facilitated their consumption, for example, washed and chopped. The vegetable item was taken from the Healthy Home Survey (item reliability, kappa = 0.57, item validity, kappa = 0.43) [29]. The reliability and validity of the Healthy Home survey items were established in a study of 85 American families with 3 to 8 year-old children by having 50% of the sample re-do the survey one week after the first administration, and by a home-visit to 95% of participants [29]. As there was no equivalent item for fruit, this was adapted from the vegetable item, specifically: *Do you have any ready to eat fresh fruit on a shelf in the refrigerator or on the kitchen counter now, for example, fruit you have washed or chopped to make ready to eat, like bunches of grapes, berries, or oranges?*

##### f) Mealtime practices

The extent to which the family was adopting mealtime practices that encouraged child fruit and vegetable consumption was measured using items from the Healthy Home Survey. Items taken from this survey included the location where most meals are eaten, the number of days per week the family sits at a table to eat dinner together, and the number of days per week the child eats dinner in front of the television (Item reliability kappa = 0.73-0.80) [29].

##### g) Family eating policies

Questions from the Healthy Home Survey were also included to assess the extent to which parents adopted eating policies that encouraged fruit and vegetable consumption. On a five-point likert scale ('all of the time' to 'never') parents recalled the frequency with which they did each of the following: ask their child to eat everything on their plate at dinner; restrict dessert if their child does not eat the food on their plate at dinner; reward their child with desserts, snacks or confectionary if they finish their dinner; allow their child to eat only at set meal times; and allow their child to help him/herself to snacks when at home (Item reliability kappa = 0.40-0.75) [29].

### Analysis

All analyses were conducted using SAS 9.2 (SAS Institute Inc., Cary, NC, USA). Descriptive statistics were used to describe the sample. Where quantitative items were used to collect information about environmental characteristics relating to fruits and vegetables separately, these totals were summed to form a single variable, for example, parental intake, parental role-modeling, fruit and vegetable availability within the home and parental providing behaviour. Similarly, the two accessibility items were combined into a single item indicating whether both fruit and vegetables were stored in a ready-to-eat format or whether fruit, or vegetables, or both fruit and vegetables were not stored in this way. Consistent with previous research on Australian parents of preschoolers [33] parental education was dichotomised into 'university educated' and 'other', and annual household income was split into less than $100,000 and $100,000 or more. Categorical variables used to assess eating policies were recoded dichotomously, whereby "all of the time" and "most of the time" responses were combined to reflect consistent adoption of these policies, and "some of the time", "rarely" and "never" were also combined. Non-normal continuous variables (days per week the family eats dinner together at a table, days per week the child eats dinner in front of the television) were treated as dichotomous categorical variables. The cut points were set at the frequencies with which the highest levels of children's fruit and vegetable consumption have previously been associated [34-37]. Therefore the number of days per week the family eats dinner together at a table was split into 7 days and less than 7 days, and the number of days per week the child eats dinner in front of the television was split into 0 days and 1 or more days. Similarly, the location where most meals were eaten was recoded into 'table' and 'other' [38].

A series of simple regression models were run investigating the association between each characteristic of the home food environment and children's fruit and vegetable consumption. Simple regression models also investigated socio-demographic characteristics for which associations with children's fruit or vegetable intake had previously been found; parental education [10], household income [39], child gender [19] and child age [19]. As numerous simple regression models were being tested, a Bonferroni adjustment was applied to the p-value (0.003) to account for the increased likelihood of type one error [40]. As evidence suggests fruit and vegetable consumption varies between children attending different childcare centres [41], all regression analyses used generalised linear mixed models (Proc Mixed) with a random intercept term to adjust for the correlation of measurements within a preschool.

A screening criterion of p < 0.25 was adopted to determine which variables would be included in the multiple regression analysis. A criterion of p < 0.25 was used as evidence suggests that adopting the traditional threshold (p < 0.05) can exclude variables of known importance [42,43]. A backwards stepwise approach was used to determine the final multiple regression model with the least significant characteristic of the home food environment removed and the analysis re-run until only significant variables remained. Socio-demographic variables that satisfied the screening criterion (p < 0.25) were controlled for in the multiple regression model (i.e. they were included in the stepwise process and retained in the final model).

## Results

The sample consisted of 396 parents, recruited from 30 preschools across the Hunter region. Of the 57 preschools within the sampling frame, 30 consented, 19 were ineligible, seven refused to participate and one could not be contacted. Children from approximately 2,200 families attended the 30 preschools, and 417 parents consented to participate, with a further 178 returning a form indicating that they did not consent to participate. Of the consenters, ten refused to participate when contacted to complete the survey, six were ineligible and five could not be contacted, resulting in a total of 396 parents providing data for the analysis. The study sample and the characteristics of their home food environments are described in Table 1.

**Table 1:** Parent, child and home food environment characteristics of the 396 study participants

Sample characteristics	Mean (SD)/%
***Parent characteristics***	
Mean age (SD) - years	35.5 (5.3)
Gender - female	96%
Aboriginal and/or Torres Strait Islander	2%
Highest educational level	
Years 7-9	2%
Years 10	11%
Year 11-12	10%
TAFE (Technical and Further Education)	30%
University	47%
Annual household income*	
< $20,000	4%
$20,000 - $39,999	9%
$40,000 - $59,999	11%
$60,000 - $79,999	15%
$80,000 - $99,999	19%
$100,000	41%

***Child characteristics***	
Mean age (SD) - years	4.3 (0.6)
Gender - female	49%
Mean daily serves of fruit (SD)^#^	2.3 (1.0)
Mean daily serves of vegetables (SD)^#^	2.1 (1.1)

***Home food environment characteristics***	
**Parental role-modeling**	
Daily serves of fruit & vegetables	5.0 (1.8)
Occasions/day modeled fruit & vegetable consumption	2.3 (1.4)
**Pressure to eat**	
Pressure to eat	3.1 (0.7)
**Parent providing behaviour**	
Times/day parent provides fruit & vegetables	3.2 (1.3)
**Fruit and vegetable availability**	
Different varieties of fruit & vegetables in home	21.7 (4.8)
**Fruit and vegetable accessibility**	
Fruit and vegetables kept in ready to eat format (% yes)	39%
**Mealtime practices**	
Always eat together as a family (7 nights per week)	57%
Never eat in front of TV (0 nights per week)	47%
Family eats most meals at table/bench (% who all or most of the time)	87%
**Family eating policies (% who all or most of the time ...)**	
Ask child to eat everything on their plate at dinner	50%
Restrict dessert if child does not eat dinner	59%
Reward with dessert if child finishes dinner	29%
Only allow child to eat at set mealtimes	39%
Allow child to help him/herself to snacks	4%

* Excluding n = 17 (don't know or refused)

^# ^Information collected from consent form

There were no significant differences between participants and those non-consenters who returned a form with respect to child age, gender, daily serves of fruit or vegetables, or level of disadvantage based on residential postcode [44]. However, only a small proportion (approximately 10%) of the families who did not participate returned a completed consent form. In comparison with a regionally representative sample of children aged 2 to 4 years, a similar proportion of children in this study consumed at least one serve of fruit per day, but a higher proportion of children in the study consumed at least two serves of vegetables per day [45].

Most parents (99%) lived with their child 7 days a week and most (74%) reported that they were 'always' responsible for their child's meals and snacks, with 22% and 5% reporting they were responsible 'most of the time' and 'half of the time' respectively. Parents consumed an average of five serves of fruit and vegetables each day and consumption levels approximated that of female adults of a similar age within the region [45]. On average, parents ate fruit and vegetables in front of their children more than two occasions per day and provided their children with fruit and vegetables more than three times a day. While, on average, households had almost 22 different types of fruit and vegetables available in the house, fewer than half of those households (39%) kept both fruit and vegetables in a ready-to-eat, accessible format. On average, families ate together at a table 5.6 days a week (with 57% eating together 7 days a week) and children ate dinner in front of the television on an average of 2.2 days a week (with 47% not doing this at all, i.e. 0 days per week). The majority of families (87%) ate most meals at a table. Although 59% of parents indicated that they would restrict dessert 'most' or 'all of the time' when their child did not eat their dinner, 29% rewarded their child with dessert for finishing dinner. Only 4% of parents allowed their child to access snacks themselves.

The mean score for the fruit and vegetable subscale for children within the study was 14.8 (sd 4.6). Table 2 displays the strength of the associations between children's fruit and vegetable score and characteristics of the home food environment and socio-demographic characteristics in simple and multiple regression models.

**Table 2:** Associations between CDQ score and characteristics of the home food environment: simple and multiple regression

	Simple Regression		Multiple Regression	
**Characteristic** **(n = 396)**	**Regression co-efficient (95% CI)**	**p-value**	**Regression co-efficient (95% CI)**	**p-value**

**Parental role-modeling**				
Daily serves of fruit & vegetables (F&V)	0.87 (0.64-1.11)	< 0.001	0.30 (0.09-0.50)	0.005
Occasions/day modeled F&V consumption	1.09 (0.78-1.40)	< 0.001		
**Pressure to eat**				
Pressure to eat	-0.78 (-1.40- -0.17)	0.012		
**Parent providing behaviour**				
Times/day parent provides F&V	2.22 (1.96-2.49)	< 0.001	1.80 (1.53-2.09)	< 0.001
**Fruit and vegetable availability**				
Different varieties of F&V in home	0.34 (0.25-0.43)	< 0.001	0.12 (0.03-0.20)	0.006
Fruit and vegetable accessibility*				
F&V kept in ready to eat format (Yes)	1.80 (0.87-2.73)	< 0.001	0.90 (0.20-1.60)	0.012
Mealtime practices*				
Always eat together as a family (7 nights per week)	0.90 (-0.02-1.82)	0.055		
Never eat dinner in front of TV (0 nights per week)	0.87 (-0.04-1.79)	0.061		
Family eats most meals at table/bench (All or most of the time)	0.48 (-0.86-1.82)	0.480		
Family eating policies*				
**(% who all of most of the time ...)**				
Ask child to eat everything on plate at dinner	-0.05 (-0.96-0.87)	0.922		
Restrict dessert if child does not eat dinner	-0.68 (-1.60-0.25)	0.151		
Reward with dessert if child finishes dinner	-0.79 (-1.79-0.21)	0.121		
Only allow child to eat at set mealtimes	1.38 (0.46-2.31)	0.003	1.00 (0.31-1.68)	0.006
Allow child to help him/herself to snacks	-1.59 (-3.90-0.72)	0.177		
**Socio-demographic characteristics^#^**				
Parental education - University	1.13 (0.22-2.05)	0.015		
Annual household income >$100,000	0.87 (-0.05-1.79)	0.065		
Child gender	0.64 (-0.27-1.55)	0.169		
Child age	-0.12 (-0.87-0.64)	0.765		

* Dichotomous characteristics

**^# ^**Parental education, household income and child gender were controlled for in the multivariate model but were not significant; p = 0.172, p = 0.848, p = 0.164 respectively.

Simple regression analysis found statistically significant positive associations (p < 0.003) between children's fruit and vegetable consumption and the following factors: parental fruit and vegetable intake; occasions per day where parents role-model fruit and vegetable consumption; provision of fruit or vegetables to children; variety of fruit and vegetables available in the home; keeping fruit and vegetables in a ready-to-eat format (e.g. washed and chopped); and only allowing children to eat at set meal times.

Twelve characteristics of the home food environment had a p-value less than 0.25 in the simple regression models and were entered into the backward stepwise regression along with parental education, household income and child gender. The assumptions of multiple regression were tested and found to be acceptable. The regression coefficients, 95% confidence intervals and p-values for the five significant variables (p < 0.05) that were retained in the final regression model are shown in the final two columns of Table 2.

Multiple regression analysis indicated that higher fruit and vegetable consumption in children was significantly associated with: higher fruit and vegetable intake in parents, more frequent provision of fruit and vegetables to children throughout the day, having a wider variety of fruits and vegetables available in the home, having fruit and vegetables stored in a ready-to-eat format, and generally only allowing children to eat at set mealtimes. These variables remained significant despite controlling for parental education, household income and the gender of the child. This model of the characteristics of the home food environments accounted for 48% of the variation in the child's fruit and vegetable score. The regression coefficients suggest that, all other factors held constant, each additional occasion that parents provide their children with fruit or vegetables throughout the day is associated with an average an increase in children's fruit and vegetable score of 1.80 points, and that ensuring that children generally only eat at set mealtimes is associated with an average increase of 1.00 points in the fruit and vegetable score. The coefficients of the remaining three significant variables within the model ranged from 0.12 to 0.90.

## Discussion

This study is one of only a handful of studies examining associations between characteristics of the home food environment and the fruit and vegetable consumption of preschool-aged children, and among the first to investigate these relationships through multiple regression analysis and with a reliable and valid measure of fruit and vegetable intake. The study found that greater fruit and vegetable consumption in children was positively associated with parent's own fruit and vegetable consumption; the frequency with which parents provide these foods to their child; the availability and accessibility of these foods in the home; and with maintaining set mealtimes. Such findings provide insights into factors that influence young children's vegetable and fruit intake.

The positive association between child and parent fruit and vegetable intake is supported by studies involving preschool-aged children [19-21] as well as older children and adolescents [14,18,48,49] and supports previous recommendations that modification of parent diet be a key strategy for interventions targeting children's eating habits [5,19,21,38,46]. A lack of significant association between child fruit and vegetable intake and parental consumption of these foods in front of their children, however, suggests that the influence of parental role-modeling is complex [47,48]. Further research investigating the mechanisms by which parental intake may influence child consumption may yield important insights for intervention.

A unique aspect of this study was the examination of parental provision of fruit and vegetables as a correlate of child consumption. Although the reported positive association is somewhat intuitive for children of this age, the finding accentuates the critical role that parents play in facilitating fruit and vegetable consumption through provision of these foods. Within the study sample at least, the findings also suggest that there is considerable scope to further improve child fruit and vegetable intake through encouraging more frequent provision. On average, parents provided fruit or vegetables to their child on 3.2 occasions per day, with dinner being the most prevalent occasion for serving vegetables, and morning tea the most prevalent occasion for serving fruit. Given that it is recommended that children of this age have three meals and two to three small snacks daily [49], introducing fruit and vegetables at additional occasions throughout the day, particularly the provision of vegetables for morning or afternoon teas, could represent an effective intervention strategy. Further, the findings of this, and other studies with older children [50,51] demonstrate a greater likelihood for children to eat fruits and vegetable if they are stored at home in a ready-to-eat form. As preparation time is a commonly cited barrier to fruit and vegetable consumption [52,53], having ready-to-eat fruit and vegetables on hand may increase the likelihood of parents feeding their preschool child these foods rather than convenient, pre-packaged, snack foods. As only 39% of parents in this study reported storing fruit *and *vegetables in this way, strategies that make it easier for parents to purchase, prepare and store ready-to-eat fruits and vegetables are needed and likely to facilitate increased parent provision of these foods to their child.

These findings should be considered in the context of the study limitations. First, this data is cross-sectional, precluding conclusions regarding causality. Further research is warranted to determine if these associations are evident in longitudinal research, and if changes to such characteristics mediate the changes to child fruit and vegetable intake following intervention. Second, use of parent volunteers may have introduced selection-bias as study participants may not be representative of the broader population from which they were drawn. Compared to a random sample of 764 mothers of 2 to 5 year-olds in the broader study region, parents in this study were more educated (47% vs 36% with a university education) and from higher income households (41% vs 20% earning over $100,000 per year) [33] and their children had higher levels of vegetable consumption than a regionally representative sample of children aged 2 to 4 years [45]. The strength of the associations found in this study is therefore unknown among families from less advantaged backgrounds. Furthermore, most participants identified themselves as the parent that was primarily responsible for feeding their child, and only 4% of the participants were fathers, most likely due to fathers being less likely to drop children at childcare [54] and being less likely to have primary responsibility for food within the household [55]. This may restrict the generalisability of study findings to mothers, and the primary food provider, rather than parents more broadly. The inclusion of measures of the home food environment with unknown validity and reliability is a further limitation of this research and further research is required to develop and refine appropriate measures suitable for population-based investigation. Finally, this research examined the combined consumption of fruit and vegetables. The analyses did not allow for the identification of the relative associations of environmental characteristics with fruit and vegetable intake separately [56]. Future research should seek to address these limitations.

## Conclusions

The study findings suggest that a range of factors within the home food environment appear to be associated with young children's fruit and vegetable intake. The final regression model which included parental intake and parental provision of fruit and vegetables to their children, the availability and accessibility of fruit and vegetables in the home and having set mealtimes accounted for almost half of the variation in children's fruit and vegetable consumption. Such results suggest that there are modifiable factors within the home environment that may be appropriate targets for future interventions aimed at increasing fruit and vegetable consumption in preschool-aged children to address this substantial public health problem.

## Competing interests

The authors declare that they have no competing interests.

## Authors' contributions

Author RW led the development of this manuscript. Authors RW, EC and LW determined the research design and the measures to be used. All authors decided upon the analyses conducted, and contributed to, read and approved the final version of this manuscript.

## Pre-publication history

The pre-publication history for this paper can be accessed here:

http://www.biomedcentral.com/1471-2458/11/938/prepub
